# Silver Nanoparticles (AgNPs) as Potential Antiviral Agents: Synthesis, Biophysical Properties, Safety, Challenges and Future Directions─Update Review

**DOI:** 10.3390/molecules30092004

**Published:** 2025-04-30

**Authors:** Abhinav Sati, Tanvi N. Ranade, Suraj N. Mali, Haya Khader Ahmad Yasin, Nehal Samdani, Nikil Navnath Satpute, Susmita Yadav, Amit P. Pratap

**Affiliations:** 1 Department of Oils, Oleochemicals and Surfactants Technology, Institute of Chemical Technology, Mumbai 400019, India; 2 Department of Pharmaceutical Chemistry, School of Pharmacy, D.Y. Patil University, Nerul, Navi Mumbai 400706, India; 3 Department of Pharmaceutical Sciences, College of Pharmacy and Health Sciences, Ajman University, Ajman P.O. Box 346, United Arab Emirates; 4 Center of Medical and Bio-Allied Health Sciences Research, Ajman University, Ajman P.O. Box 346, United Arab Emirates; 5 Department of Pharmaceutical Sciences and Technology, Birla Institute of Technology, Mesra 835215, India

**Keywords:** silver, silver nanoparticles, antiviral, antiviral mechanism, virucidal, applications

## Abstract

AgNPs have gained significant attention due to their unique physicochemical properties, making them valuable across a range of fields including medicine, textiles, and household products. With their strong antimicrobial and antiviral properties, AgNPs have shown promise in treating infections, particularly in wound care management. This review explores the mechanisms underlying the antiviral activities of AgNPs, as well as the methods used for their synthesis, which include physical, chemical, and biological approaches. The review also addresses the potential limitations of AgNPs, including their cytotoxicity to humans and the environment. The interaction between AgNPs and microorganisms, particularly viruses, varies based on synthesis methods and particle morphology. As viral infections, including resistant strains, present major global health challenges, there is a growing need for alternative antiviral therapies. Metal nanoparticles like AgNPs offer potential advantages over conventional antiviral drugs due to their broad target range, which reduces the likelihood of resistance development. This review highlights AgNPs’ effectiveness against a variety of viruses, such as HIV, hepatitis B, and respiratory syncytial virus, and discusses their potential for use in novel antiviral treatments. The review also examines AgNPs’ toxicity, offering insights into their future therapeutic roles.

## 1. Introduction

Viruses emerge due to changes in hosts, environments, or vectors, with several becoming widespread in humans, such as SARS, Nipah, and Chikungunya. Despite advances in detection, prevention, and treatment lag behind, with antiviral resistance posing challenges. New and recurring viruses continue to threaten public health, infecting humans, animals, and plants, leading to disease outbreaks, economic strain, and loss of life. Nanotechnology, an interdisciplinary field, offers promising solutions, particularly through metal-based nanoparticles with unique physico-chemical properties [1,2,3]. Their controlled size, shape, and surface area enable antiviral applications. While widely used in electronics and materials science, nanotechnology’s role in medicine is rapidly expanding, opening new avenues for antiviral development and disease control [4,5]. Nonetheless, the list of viral diseases for which antiviral therapies or vaccines are available is still relatively short and some viruses are developing resistance to current therapies [6]. Over the past ten years, nanotechnology has emerged as a promising approach to combat viruses. AgNPs, known for their broad-spectrum antimicrobial properties, have gained significant interest in the scientific community [7].

AgNPs exhibit antimicrobial activity in many ways and there are multiple mechanisms reported so far in the literature [6]. These mechanisms rely on multiple factors such as size, shape of nanoparticles, pH, ionic strength of media, etc. Moreover, the role of the capping agent has also proven a crucial factor. The release of the silver ions (Ag^+^) from AgNPs is considered a prominent mechanism behind the antimicrobial potential of AgNPs. The positively charged silver ions, in particular, are crucial for antibacterial activities and should remain in the same ionized state. Moreover, such charged ions form complexes with nucleic acids and thus, interact with nucleosides. In some cases, negatively charged cells were also attracted towards Ag^+^ from AgNPs. Literature is also available stating the adherence of Ag^+^ ions to the cytoplasm, due to affinity towards such sulfur proteins. Moreover, such free ions tend to deactivate the respiratory enzymes in the cells, giving rise to reactive oxygen species (ROS). The generations of ROS lead to DNA damage. Furthermore, it was also noted that such ROS stops the protein synthesis mechanisms. Another mechanism could also include the modulation of cellular signaling. Apart from ions, AgNPs themselves brings protein denaturation leading to microbial deaths. However, antimicrobial resistance to silver NPs is a key limitation.

Variations in chemical composition, morphology, particle size, and dispersity play a crucial role in determining the physicochemical properties of nanoparticles (NPs). These variations mainly arise from the synthesis process, which is governed by several key factors. Contemporary nanoparticle production methods focus not only on attaining accurate nanoscale dimensions but also on maintaining efficiency, cost-effectiveness, environmental sustainability, and suitability for diverse industrial and scientific applications [8,9,10,11]. Their potential applications span various biomedical fields, with sizes typically ranging from 1 to 100 nanometers [12]. There are numerous techniques available for synthesizing AgNPs, including chemical, physical, and biological methods, each of which can greatly impact their size, structure, and characteristics. However, various parameters such as dispersing agents, surfactants, and temperature can be regulated to produce AgNPs with specific sizes and properties [12]. AgNPs demonstrate significant antiviral capabilities by engaging with viral particles and preventing their replication. Their effectiveness stems from their surface characteristics, enabling them to bind to viral membranes and disrupt their structural stability. Studies have shown that AgNPs can effectively neutralize viruses like influenza and herpes simplex by interfering with viral proteins and inhibiting their entry into host cells. However, further research is necessary to confirm their safety for use in biological systems [13].

## 2. Synthesis

**Figure 1:**
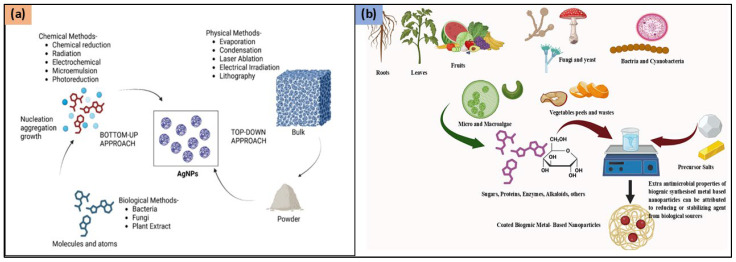
Synthesis methods for AgNPs (**a**) Chemical, Physical, and Biological Methods and (**b**) Various biological sources for ‘Green synthesis’ of AgNPs. Figure 1a reproduced from Ref. [14], Copyright 2025, American Chemical Society.

Physical, chemical, and biological approaches can be utilized for the synthesis of AgNPs (Figure 1). The process of synthesizing AgNPs includes the reduction of silver ions (Ag^+^) into metallic silver (Ag⁰), followed by nucleation and subsequent growth, influencing nanoparticle size, shape, and properties. The chosen synthesis method impacts the morphology and characteristics of the resulting AgNPs.

### 2.1. Physical Methods

**Figure 2:**
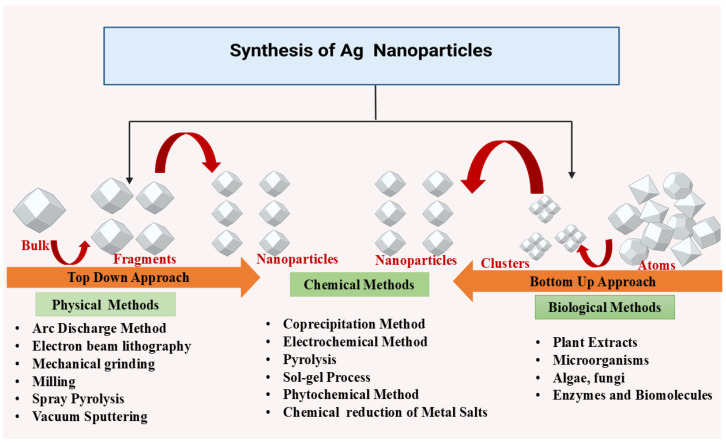
Different methods of synthesizing AgNPs.

Physical methods utilize external forces to produce AgNPs from bulk silver (Figure 2). Techniques such as milling, arc discharge, and laser ablation generate nanoparticles with uniform size, high purity, and controlled morphology [14,15]. Ball milling involves mixing metal particles with milling balls in a medium of gas (e.g., inert gas or air). Parameters like milling time, rotational speed, and temperature influence particle morphology. Larger particles tend to agglomerate due to reduced surface energy, while temperature affects phase characteristics. In a study conducted by Khayati and Janghorban, nanostructured silver with a crystallite size of 28 nm was synthesized using a high-energy ball mill, followed by the reduction of Ag_2_O with graphite [16]. The Electrical Arc-Discharge Method utilizes a DC power source applied between silver electrodes immersed in a dielectric liquid [17]. The high temperature vaporizes silver, which then condenses into AgNPs [18]. Elwakil et al. synthesized carbon-coated AgNPs (17 nm), exhibiting strong antibacterial activity against *Pseudomonas aeruginosa* and cytotoxicity against normal lung cells [19]. In another study, Gharieb et al. synthesized AgNPs-CNTs (9 ± 2 nm) at −10 °C and AgNPs/C (12.4 ± 3 nm) at 25–55 °C using ethanol as a dielectric medium [20]. In the Laser Ablation Method, a pulsed laser targets bulk silver within a liquid medium, producing plasma plumes that cool and form nanoparticles [21,22]. The characteristics of the nanoparticles are influenced by factors such as laser fluence, pulse duration, and the type of solvent used. Rahmah et al. synthesized spherical AgNPs (~30 nm) with a band gap of 2.25 eV, showing anticancer activity and antibacterial effects [23]. Rafque et al. used a one-step green laser ablation method, producing smaller nanoparticles (9 nm) with enhanced thermal conductivity [24]. Kenmotsu et al. synthesized AgNPs for SERS applications using laser ablation, post-annealing, and electrostatic mobility classification [25]. Mohammed et al. conjugated AgNPs with *Elettaria cardamomum* seed extract, enhancing antibacterial efficacy against *Escherichia coli* and *Staphylococcus aureus* using laser ablation techniques [26]. Niaz et al. investigated the effect of confinement geometry on pulsed laser ablation [27]. Alharbi et al. combined laser ablation with RF sputtering to produce ZnO-encapsulated AgNPs, improving photodetector efficiency [28]. Raffi et al. synthesized AgNPs (8–32 nm) using inert gas condensation, demonstrating size control through evaporation temperature and helium pressure [29].

### 2.2. Chemical Methods

Chemical methods such as chemical reduction, are used for AgNP synthesis. These processes involve reducing the silver ions (Ag^+^) into metallic silver (Ag^0^) using organic or inorganic reducing agents (Figure 2). The resulting silver clusters exhibit a characteristic color due to surface plasmon resonance. Alternative chemical methods include microemulsion techniques and microwave-assisted synthesis, offering efficient control over nanoparticle size and properties [30,31,32]. Chemical reduction utilizes a reducing agent and a silver precursor, typically silver nitrate, to produce AgNPs. Sodium borohydride is a common reducing agent, often combined with stabilizers like trisodium citrate to control nanoparticle nucleation and growth. Agnihotri et al. synthesized AgNPs ranging from 5 to 100 nm with monodisperse characteristics using sodium borohydride and trisodium citrate [33]. The choice of stabilizing agents influences nanoparticle morphology, allowing the formation of alternative structures like rhomboidal particles and nanosheets [34]. Microemulsion synthesis involves surfactants to mix immiscible liquids, such as oil and water, enabling the production of uniform AgNPs. Reactants are separated into two phases, with the interfacial region facilitating controlled nanoparticle growth. Surfactants like sodium dodecylbenzene sulfonate, cetyltrimethylammonium bromide (CTAB), and polyvinylpyrrolidone (PVP) determine AgNP characteristics [8,35,36]. Hak et al. demonstrated the use of microemulsions for targeted breast cancer therapy, achieving controlled drug release and enhanced bioavailability in MCF-7 cells [37]. Eco-friendly AgNP synthesis can be achieved using polysaccharides and polymers as reducing and capping agents. Starch-based AgNPs, synthesized with *D*-glucose as a reducing agent, exhibit thermal reversibility due to weak starch-nanoparticle interactions. Other reducing agents include polyethylene glycol (PEG), polyvinyl alcohol (PVA), and chitosan with PEG-coated AgNPs demonstrating good stability in high saline solutions [38,39,40,41,42]. Singh et al. incorporated AgNPs into hydrogels using tragacanth gum and gum acacia, leading to sustained drug release and antimicrobial properties. Sarkar et al. synthesized AgNPs using bacterial polysaccharides, achieving significant photocatalytic degradation of azo dyes, highlighting AgNPs’ potential in bioremediation [43]. Chemical synthesis methods offer precise control over AgNP properties, making them valuable for applications in medicine, drug delivery, and environmental remediation.

### 2.3. Green Synthesis

Sustainability in silver nanoparticle (AgNP) synthesis has become increasingly important, as chemical and physical methods often leave behind harmful solvent residues like ethylene glycol, sodium citrate, oleyl amine, and liquid paraffin (Figure 2). Such residues pose risks, particularly in applications involving drug delivery, antimicrobial treatments, and human exposure. Green synthesis, an eco-friendly alternative, relies on natural sources such as microorganisms (fungi, yeasts, bacteria, and actinomycetes), and plant extracts. This method has gained traction across various sectors, including biomedicine, cosmetics, food, drug delivery, and agrochemicals, due to its sustainable approach and the unique properties of AgNPs. The green synthesis of AgNPs primarily requires a solution of silver metal ions and a biological reducing agent (Table 1). In most cases, the constituents within the cells act as natural stabilizing and capping agents, making the addition of external stabilizers unnecessary. Reducing agents are naturally present in various biological systems. AgNPs have been synthesized using organisms from four of the five kingdoms of life: Monera (prokaryotic organisms lacking a true nucleus), Protista (unicellular eukaryotic organisms with a true nucleus), Fungi (eukaryotic organisms that are either saprophytic or parasitic), Plantae (eukaryotic autotrophs), and other Animal-derived materials (eukaryotic heterotrophs) [44,45]. Ahmed et al. studied a rapid, eco-friendly synthesis of AgNPs using *Azadirachta indica* leaf extract as both a reducing and capping agent. Characterized by FTIR, DLS, TEM, and UV-Vis (436–446 nm), the nanoparticles showed strong antibacterial activity against *Escherichia coli* and *Staphylococcus aureus*. The one-pot green synthesis is cost-effective, stable, and excludes hazardous chemicals, making it a viable alternative to conventional methods for biomedical and optoelectronic applications [46]. In another study by Ashraf et al. the anti-glycating potential of AgNPs synthesized using *Aloe vera* extract was studied. Characterization through UV-Vis, EDX, TEM, XRD, and DLS confirmed that AgNPs (~30.5 nm) significantly inhibited AGE formation in a concentration-dependent manner while preserving protein structure. The study emphasizes their potential therapeutic applications in diabetes-related complications, antimicrobial activity, and antiviral effects [47]. Das et al. synthesized AgNPs using *Trema orientalis* leaf extract as a reducing and stabilizing agent. Characterization via UV-Vis, FTIR, TEM, XRD, and AFM confirmed their crystalline nature with sizes ranging from 14.04 to 34.38 nm. AgNPs demonstrated significant antibacterial activity against *Staphylococcus aureus*, with inhibition zones increasing at higher concentrations. This eco-friendly synthesis method offers a sustainable approach to developing antimicrobial agents with potential biomedical applications [48]. Singla et al. investigated the synthesis of AgNPs using *Oxalis griffithii* methanolic leaf. The AgNPs exhibited a spherical, crystalline structure with stability confirmed by Zeta potential. UV-Vis absorption peak appeared at 408–412 nm. The antibacterial activity was tested against *Escherichia coli* and *Bacillus subtilis*, demonstrating significant effectiveness. This eco-friendly synthesis method offers a cost-effective approach for potential biomedical applications [49]. In another study by Widatalla et al., green tea (*Camellia sinensis*) leaf extract was utilized as a natural reducing agent for the eco-friendly synthesis of AgNPs. UV-Vis spectroscopy confirmed their formation with a peak at 410 nm, while SEM analysis revealed sizes ranging from 15 to 33 nm. FTIR analysis identified polyphenols, polysaccharides, and proteins involved in the synthesis. Antibacterial studies demonstrated strong inhibition against *Staphylococcus aureus* and *Klebsiella* sp., highlighting their potential as antimicrobial agents [50]. Khane et al. [50], used *Citrus limon* zest extract as a natural reducing agent for the eco-friendly synthesis of AgNPs. Characterization through UV-Vis, FTIR, SEM, TEM, XRD, and DLS confirmed their formation, with a surface plasmon resonance peak at 535.5 nm. The crystalline, spherical AgNPs exhibited strong antimicrobial effects against *E. coli*, *Staphylococcus aureus*, and *Candida albicans*, along with significant antioxidant properties, highlighting their potential applications in biomedical and pharmaceutical fields.

## 3. Biophysical Properties

Nanomaterials possess unique properties distinct from their bulk counterparts, influenced by chemical composition, morphology, and surface structure. Their small size leads to enhanced reactivity, optical, and mechanical characteristics. These attributes make them valuable in diverse applications, including targeted drug delivery, bio-imaging, and disease diagnosis. Their high surface area and tunable properties enable advancements in medicine, electronics, and environmental science, driving innovation in multiple fields [58,59]. The properties of silver nanoparticles are outlined below:

### 3.1. Shape and Crystallinity

Various synthesis techniques enable the production of AgNPs in diverse shapes and sizes, including nanospheres, nanorods, nanobars, nanoprisms, decahedral nanoparticles, and triangular bipyramids [60]. For instance, a photo-induced approach has been employed to convert spherical AgNPs into triangular nanoprisms [61,62,63]. Research by Mirkin and Murphy investigated a seeding method for synthesizing Ag nanoprisms with controlled edge lengths [64,65]. Another study introduced a modified polyol process where ethylene glycol functions as both a solvent and a reducing agent, yielding AgNPs in different forms such as pentagonal nanowires, right bipyramids, and nano-cubes with adjustable corner truncation. Additionally, microwave heating has become a widely used method for synthesizing triangular Ag nano-plates [66,67,68,69,70]. The scanning electron microscopy (SEM) and X-ray diffraction (XRD) analyses confirmed the crystalline structure and morphological characteristics of the AgNPs, which exhibited both spherical and hexagonal geometries [71]. The morphological characteristics, dimensional parameters, and structural attributes of the synthesized nanoparticles were analyzed using scanning electron microscopy (SEM), UV-visible spectroscopy (UV-VIS), and X-ray diffraction (XRD) techniques. SEM imaging revealed that the AgNPs exhibited a predominantly spherical shape with diameters ranging from 15 to 90 nm, while triangular nanoparticles possessed edge lengths of approximately 150 nm. UV-VIS spectroscopy indicated that the surface plasmon resonance (SPR) peaks of the spherical silver colloids appeared within the 397–504 nm wavelength range. In contrast, the triangular nanoparticles displayed two distinct SPR peaks, one at 392 nm and another at 789 nm [72]. Triangular or rod-shaped AgNPs can enhance plasmonic and antibacterial effects due to their anisotropic shapes, but their higher cytotoxicity, aggregation tendencies, and limited biocompatibility often restrict their biomedical applications. For a wide range of biomedical applications, silver nanoparticles (AgNPs) in the size range of 10–30 nm are generally considered optimal, offering a good balance between antimicrobial efficacy and biocompatibility. Smaller nanoparticles (approximately 5–15 nm) are particularly advantageous for cell membrane penetration and targeted drug delivery, while larger particles (around 40–60 nm) may be better suited for applications such as wound healing and tissue regeneration, where slower release and surface interactions are beneficial [73,74].

### 3.2. Melting Temperature

Thermal behavior is a crucial factor in the production and application of materials. A notable characteristic of metal nanoparticles is their reduced melting temperature, attributed to the thermodynamic size effect, which has been widely utilized for various applications. The thermal properties of AgNPs are commonly analyzed using thermogravimetric analysis (TGA) or differential scanning calorimetry (DSC). Additionally, the Gibbs–Thomson equation serves as a theoretical approach to studying the thermal behavior of nanoparticles [75]. Metallic nanoparticles exhibit significantly lower melting points than their bulk counterparts [76]. For example, while bulk silver has a fixed melting point of 960 °C, AgNPs demonstrate a lower and size-dependent melting temperature. This behavior can be explained by the Gibbs−Thomson effect, which describes how a higher surface area-to-volume ratio in smaller nanoparticles contributes to increased surface energy [77]. Consequently, there is an inverse relationship between nanoparticle size and melting point. The Gibbs−Thomson effect also explains the tendency of smaller nanoparticles to undergo sintering or Ostwald ripening, reducing their total free energy and resulting in surface melting at lower temperatures [78,79].

### 3.3. Optical Properties

The optical properties of metal nanoparticles have been a significant area of study in physical chemistry since the 19th century. With advancements in lithographic techniques and wet chemistry, it is now possible to synthesize noble metal nanoparticles in a variety of sizes, shapes, and dielectric environments. Understanding their interaction with light requires solving Maxwell’s equations for light scattering, especially for particles with complex geometries. Key aspects include dipole and quadrupole plasmon resonances in spherical nanoparticles, as well as analytical and numerical methods for determining extinction and scattering cross-sections, local field distributions, and other optical characteristics in nonspherical structures. These theoretical approaches play a crucial role in exploring the behavior of triangular AgNPs and related morphologies, which are of growing interest in various applications [62]. The optical activity of AgNPs was investigated by experimentally measuring extinction, scattering, absorption cross-sections, and efficiencies in water. Measurements were conducted for 16 distinct particle sizes, ranging from 29 to 136 nm, under chemically clean conditions. Results revealed that these nanoparticles interact with light 4 to 10 times more intensely than predicted by their geometric cross-section. Additionally, the absorption and scattering contributions to the plasmon resonance were independently analyzed across the visible spectrum. To facilitate the accurate determination of particle concentrations, regardless of variations in size, shape, or aggregation state, a method called standard subtraction was introduced, offering a simple and reliable approach [80]. In metallic silver, light interaction occurs due to the confinement of conduction electrons within nanoscale dimensions and the frequency-dependent dielectric function. These factors contribute to the phenomenon of surface plasmon resonance (SPR), where the collective oscillation of conduction electrons is induced by an external electromagnetic field [61]. Numerous studies demonstrated that AgNPs absorb electromagnetic radiation within the visible spectrum, specifically in the 380–450 nm range, through a mechanism known as localized surface plasmon resonance (LSPR) excitation. The study of the optical response of embedded AgNPs reveals a strong dependence of surface plasmon resonances (SPRs) on nanoparticle morphology and dielectric environment. Using the discrete dipole approximation, extinction efficiencies were analyzed for nanoparticles of varying shapes and ambient conditions. The findings indicated that an increase in truncation leads to a blue shift in the primary SPR, with overlapping resonances at shorter wavelengths and broadening of the main resonance. As the number of facets increases, SPRs diminish, particularly when nanoparticle symmetry is enhanced. Conversely, sharper vertices result in a greater number of distinct SPRs. Environmental refractive index variations do not alter the number of SPRs but significantly influence their spectral position and width, with higher refractive indices inducing a redshift. These results provide crucial insights into the tunability of plasmonic properties for applications in sensing, imaging, and nanophotonics [81].

### 3.4. Electrical Properties

The electrical performance of low-temperature screen-printed AgNPs (nAg) has been evaluated at frequencies up to 220 GHz, revealing superior characteristics compared to conventional thick-film silver conductors. Notably, for frequencies above 80 GHz, coplanar waveguide structures fabricated with nAg at 350 °C exhibit lower electrical losses than those composed of micrometer-sized grains sintered at 850 °C. This enhancement is attributed to improved nanoparticle packing, leading to a threefold reduction in surface roughness. These findings demonstrate the potential of AgNPs for high-frequency applications, particularly on temperature-sensitive conformal substrates and in sub-THz metamaterials, offering new possibilities for advanced electronic and photonic devices [82]. Electrical characterization revealed that the current exhibited an increasing trend with the enhancement of the nanoparticles’ antioxidant properties. Consequently, the antioxidant potential of the synthesized nanoparticles can be estimated through an analysis of their electrical characteristics [83]. The silver nanoparticle (AgNP)-loaded silk films exhibit flexibility and demonstrate significant variations in electrical conductivity, making them highly suitable for applications in biosensors and implantable thermoelectric wireless switching devices [84].

## 4. An Update on Antiviral AgNPs

**Figure 3:**
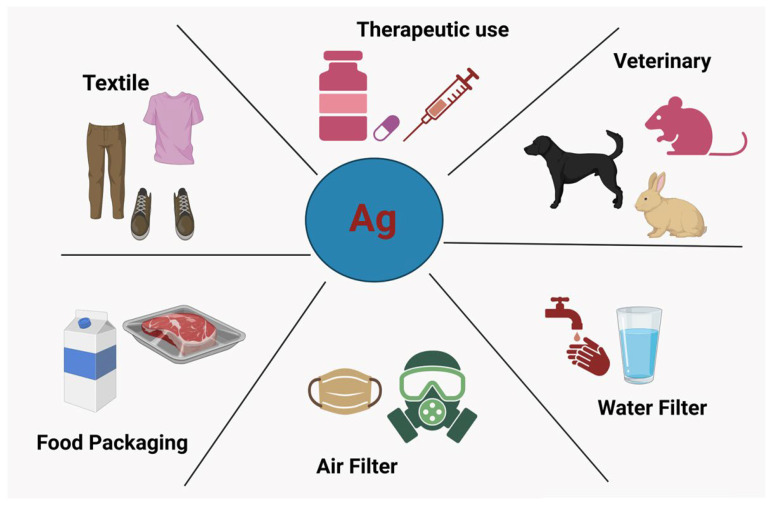
Applications of AgNPs in the field of virology. accessed on 1 January 2025, Adapted from open access article under the Creative Commons Attribution (CC BY) license (https://creativecommons.org/licenses/by/4.0/). Luceri, A.; Francese, R.; Lembo, D.; Ferraris, M.; Balagna, C. Silver Nanoparticles: Review of Antiviral Properties, Mechanism of Action and Applications. *Microorganisms* **2023**, *11*, 629. https://doi.org/10.3390/microorganisms11030629 [87].

Recent advancements highlight AgNPs as promising antiviral agents due to their broad-spectrum activity (Table 2, Figure 3). Their effectiveness depends on key factors like size (optimal at ~10 nm), concentration, and functionalization, which can enhance virus interaction or block host cell entry. Despite their potential for air/water purification, textiles, and biomedical applications, commercial adoption remains limited. Future research should focus on optimizing antiviral mechanisms, improving safety, and developing sustainable recycling methods to minimize environmental impact while enhancing efficacy. Additionally, AgNPs have demonstrated antiviral efficacy against a range of viruses, including HIV, hepatitis B, and coronaviruses, by interfering with viral entry, replication, and protein interactions. Their nanoscale properties enable them to attach to viral envelopes and disrupt structural integrity, reducing infectivity. While their potential in antiviral therapy is evident, optimizing synthesis methods, improving targeted delivery, and mitigating cytotoxicity remain critical challenges. Ongoing research aims to refine AgNP formulations for safer and more effective clinical applications [58]. Viral infections continue to pose significant global health challenges, with conventional antiviral agents often associated with toxicity and resistance issues [85]. AgNPs have emerged as promising antiviral agents due to their broad-spectrum activity against various pathogenic viruses. Their antiviral efficacy is influenced by synthesis methods, which dictate the size, morphology, and surface charge. Biogenic and hybrid synthesis approaches offer advantages such as enhanced stability, reduced toxicity, and synergistic antiviral effects, making AgNPs a potential alternative for combating viral infections [86].

There has been research which highlights the the potential of metal-based nanocomposites, particularly silver-containing polyelectrolyte complexes, in antiviral applications. These nanocomposites, stabilized by natural polymers like chitosan and pectin, exhibit potent antimicrobial and antiviral properties. It also indicated their effectiveness against herpes simplex virus type 1 and influenza virus, with no observed cytotoxic effects on MDCK and BHK cells. Additionally, ultraviolet-induced reduction of silver ions enhances nanoparticle formation, influencing their antiviral efficacy. These findings support the development of silver-based nanomaterials for biomedical use [111]. A study by Sinclair et al. highlighted that the antiviral activity of silver nanoparticles (AgNPs) is strongly influenced by surface charge, more than size, shape, or concentration. Various capping agents imparting different charges were tested, with BPEI-capped AgNPs showing the highest antiviral effect against MS2 bacteriophages due to strong electrostatic attraction. TEM analysis confirmed structural damage to the virus, suggesting that positively charged coatings enhance AgNP efficiency, enabling lower dosages for antiviral applications [112]. In a study by Bharti et al., trisodium citrate was used as a capping agent to stabilize silver nanoparticles (AgNPs), enhancing their antiviral efficacy. Capping agents prevent nanoparticle agglomeration, maintain optimal size (1–10 nm), and promote effective interaction with viral proteins. Other agents like PVP and BSA have shown similar effects, influencing surface chemistry and antiviral mechanisms such as capsid binding, replication inhibition, and reduced silver ion leaching [113]. Souza et al., investigated the anti-SARS-CoV-2 activity of silver nanoparticles (AgNPs) capped with oleylamine (OAm). Spherical, hydrophobic nanoparticles (8 ± 2 nm) were deposited on surgical mask textiles, showing up to 99.6% virus inactivation within 2 min. In contrast, free OAm exhibited 67% activity after 10 min. Low nanoparticle leaching was observed in contact with water or culture medium, emphasizing the importance of capping molecules in designing effective, long-lasting anti-SARS-CoV-2 coatings [89]. Another study by Emam et al. investigated the use of crocin as a capping agent in the synthesis of silver nanoparticles (Ag NPs), which were then incorporated into polyacrylonitrile (PAN) nanofibers for antiviral protection. The crocin-capped Ag NPs, with a size of 23.21 nm, showed good biocompatibility and effective inhibition of the ACE2-SARS-CoV-2 spike protein interaction. The Ag-PAN nanofibers demonstrated strong antiviral activity, highlighting the importance of capping agents in enhancing the antiviral properties of Ag NPs [114]. Recent advancements in nanotechnology have led to the development of various functional nanoparticles with promising antiviral properties, including quantum dots, gold and AgNPs, graphene oxide, and dendrimers. These nanostructures exhibit antiviral efficacy through diverse mechanisms, offering potential for targeted drug delivery. However, challenges remain in improving biocompatibility, optimizing drug release, and addressing nanoparticle toxicity. Further research is needed to refine antiviral nanodrugs, enhance their specificity, and develop safer formulations for clinical applications [115]. Similarly, Park et. al, in their study explored silver nanoparticle-magnetic hybrid colloids (AgNP-MHCs) as an antiviral solution with minimal environmental risks. AgNP-MHCs effectively inactivated bacteriophage X174 and murine norovirus (MNV) through surface interactions, while adenovirus serotype 2 (AdV2) remained resistant due to its size and structural properties. The study highlighted the role of AgNP size, silver concentration, and surface area in antiviral efficacy. Additionally, AgNP-MHCs maintained performance under varying pH and water conditions and were recoverable via magnetism, reducing environmental concerns [116]. Furthermore, Paulina et. al, studied the increasing demand for antiviral strategies, especially in response to the COVID-19 pandemic, highlighting advancements in antiviral surface materials and coatings. Various materials, including metallic nanoparticles (AgNPs, CuO, ZnO), nanofibrous membranes, and biopolymer-based films, have demonstrated virus-inhibiting properties. The effectiveness of these materials depends on their physicochemical attributes, influencing virus attachment and persistence. However, challenges remain in standardizing testing protocols and assessing environmental impacts, emphasizing the need for systematic studies to optimize antiviral strategies for public health applications [117]. Pathogens cause severe infections and global mortality, necessitating advanced antiviral strategies. Samreen et. al, work highlighted AgNPs as potent antiviral agents due to their unique physicochemical properties, enabling them to disrupt viral structures, inhibit replication, and enhance immune response. Their high surface reactivity allows efficient virus binding, reducing infectivity. Moreover, AgNP-based nanocarriers enhance drug delivery, overcoming resistance issues. However, challenges like toxicity, stability, and cost require further research to optimize their clinical application [118]. Similarly, infectious diseases contribute to over 20% of global mortality, with viral infections accounting for a significant portion. The rapid emergence of highly contagious viruses, such as SARS, MERS, and COVID-19, underscores the urgent need for antiviral solutions. While conventional antiviral drugs exist, their efficacy is often limited, and resistance remains a concern. Nanoparticles, particularly AgNPs, offer promising antiviral potential due to their unique physicochemical properties. They can inhibit viral replication, disrupt viral envelopes, and enhance drug delivery, making them a viable alternative for combating viral infections, including SARS-CoV-2 [119]. Mofida et. al, explored the synthesis of AgNPs using Ulva lactuca, assessing their antiviral and antioxidant potential. Characterization confirmed the formation of spherical nanoparticles (4.08–27.57 nm, average 10.29 nm). While these AgNPs demonstrated strong antioxidant activity, they exhibited limited antiviral effects against Adenovirus. Optimization parameters such as reaction time, precursor concentration, and pH were evaluated. The antioxidant mechanism was attributed to functional groups from the algal extract, influencing stability and reactivity, aligning with previous findings on marine-derived AgNPs [106].

### AgNPs Exhibit Antiviral Activity-Mechanisms Behind

**Figure 4:**
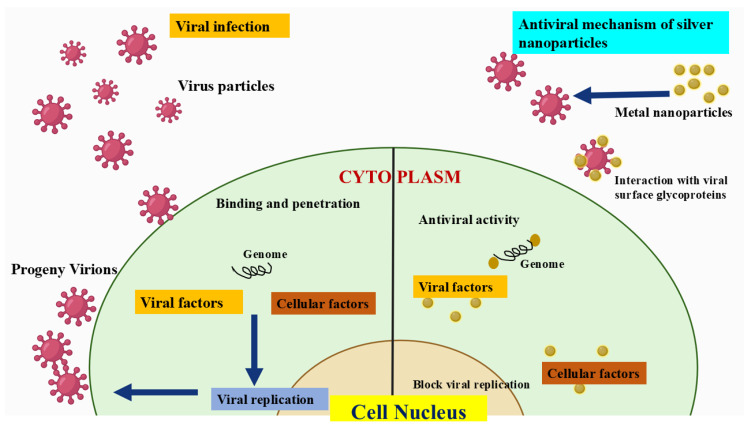
The antiviral activities of AgNPs-Mechanism. Adapted from Ref. [115], Copyright 2011, MDPI.

AgNPs exhibit antiviral activity through multiple mechanisms. They can directly interact with viral surface proteins, preventing attachment and entry into host cells. AgNPs also disrupt viral integrity by damaging the viral envelope or capsid, leading to inactivation. Additionally, they induce reactive oxygen species (ROS) generation, causing oxidative stress that harms viral components. Another key mechanism involves AgNPs binding to viral RNA or DNA, interfering with replication and transcription (Figure 4). These combined actions make AgNPs promising candidates for antiviral therapies. Viral attachment and entry are one of the primary ways AgNPs inhibit viral infections. The viral invasion begins when the virus gets bound to specific receptors on the cell membrane of the host. AgNPs can attach to the viral surface proteins, thereby altering their conformation and preventing successful receptor recognition. This type of mechanism has been observed in herpesviruses, where AgNPs effectively block glycoprotein-mediated binding, and reduce infection rates [120]. Another vital antiviral property of AgNPs, is their ability to disrupt the structural integrity of viral particles. They interact with viral envelopes or capsids, and induce morphological changes that compromise the stability of viral particles. This action of structural disruption prevents viral fusion with host cell membranes, rendering the virus non-infectious. Moreover, AgNPs induce oxidative stress through ROS generation, thereby leading to extensive damage to viral proteins, lipids, and nucleic acids. The oxidative stress caused by AgNPs is particularly detrimental to enveloped viruses, as it disrupts lipid bilayers and essential proteins required for viral replication. This mechanism contributes to the broad-spectrum antiviral potential of AgNPs [87]. AgNPs also play a significant role in inhibiting viral replication and transcription. By binding to viral RNA or DNA, they obstruct key processes necessary for viral propagation.

**Figure 5:**
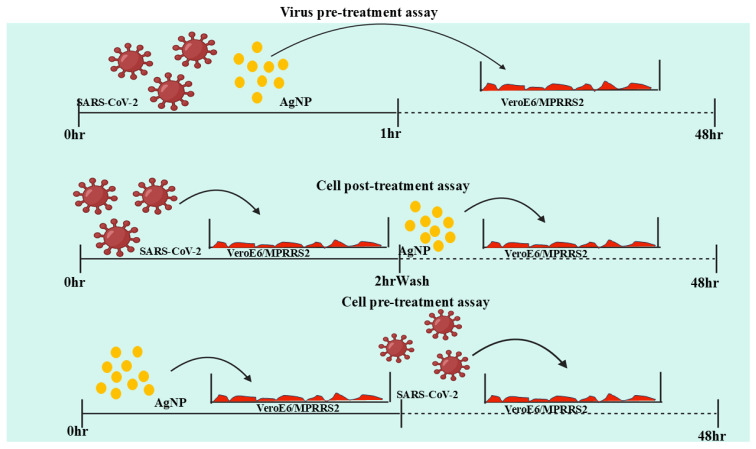
Action of AgNPs on SARS-CoV-2 virus.

In RNA viruses, AgNPs interfere with polymerase enzymes, preventing the synthesis of new viral genomes. In DNA viruses, they can disrupt the replication machinery, inhibiting the production of viral progeny. This interference effectively halts the viral life cycle and limits the spread of infection [121]. Additionally, AgNPs can modulate the host immune response, enhancing antiviral defenses. They have been shown to stimulate cytokine production and activate immune pathways that strengthen the host’s ability to combat viral infections. This immunomodulatory effect further supports their therapeutic potential [122]. These multifaceted antiviral mechanisms make AgNPs highly effective against a range of viruses, including herpesviruses, coronaviruses, influenza viruses, and others. Their broad-spectrum activity, combined with their ability to target multiple stages of the viral life cycle, highlights their potential for innovative antiviral treatments. However, further research is needed to optimize their application, ensuring maximum efficacy while minimizing potential cytotoxic effects on host cells [123]. AgNPs exhibit antiviral properties by interfering with viral attachment, particularly against herpesviruses. They can bind to viral surface proteins, preventing the virus from attaching to and entering host cells. This disruption in the initial stages of infection reduces viral spread and replication. Due to their nanoscale size and unique physicochemical properties, AgNPs offer a promising approach to antiviral therapies by targeting viral-host interactions at the molecular level [124]. Thibault et. al, research highlights AgNPs’ potential in veterinary medicine for infection control, including their role in preventing viral diseases. Studies suggested that AgNPs could serve as alternatives to conventional antivirals, reducing reliance on antibiotics [125]. Similarly, the emergence of SARS-CoV-2 highlighted gaps in CoV prevention, while swine CoVs continue to pose economic threats. Recent research explored chitosan-modified silver nanoparticles (Chi-AgNPs) for their antiviral potential. In vitro studies demonstrated that Chitosan-AgNPs disrupt viral entry by altering the spike protein’s secondary structure through disulfide bond cleavage. Additionally, they inhibit virus-induced apoptosis in Vero cells via ROS/p53 signaling. These findings suggest Chi-AgNPs as a promising preventive strategy against CoV infections [126]. In Qinghao et. al, research AgNPs have demonstrated potent virucidal effects, with their efficacy varying based on surface modifications and particle size. Among tested AgNPs, 50-nm BPEI showed the strongest antiviral activity. Studies also confirmed AgNPs’ relative safety compared to silver ions, making them promising for transmission prevention (e.g., PPE coatings, nasal rinses) and therapeutic use. However, further research is essential to explore their full potential as broad-spectrum virucides [127]. Sangiliyandi et. al, in her work, studied that infectious diseases contribute to significant global mortality, with viral infections being a major cause. The frequent emergence of highly infectious viruses like SARS, MERS, and COVID-19 poses severe threats to public health and economies. Figure 5 shows a schematic representation of how AgNPs are effectively used in inhibiting SARS-CoV-2 viruses.

Despite ongoing research, there are limited antiviral drugs and vaccines available, necessitating alternative strategies [128]. Nanoparticles offer a promising approach due to their unique properties, including antiviral activity, low resistance potential, and biocompatibility. nanomaterials like silver, gold, quantum dots, liposomes, and polymers for antiviral applications, focusing on their mechanisms, therapeutic potential. Similarly, the antiviral potential of tannic acid-modified AgNPs against HSV-2. AgNPs (13–46 nm) exhibited size-dependent antiviral activity by directly inhibiting virus attachment, penetration, and spread, both in vitro and in vivo. Smaller AgNPs induced cytokine production, enhancing the antiviral response. Unlike tannic acid alone, AgNPs effectively reduced infection and inflammation in a mouse model. Given the structural similarity of HSV-1 and HSV-2, these nanoparticles could serve as topical microbicides for treating both oral and anogenital herpes [124]. Polyvinylpyrrolidone (PVP)-coated AgNPs ranging from 1–10 nm in size have demonstrated antiviral activity against HIV-1 (Human Immunodeficiency Virus Type 1). These nanoparticles interact with gp120, a glycoprotein present on the surface of the HIV-1 virus, which is crucial for viral attachment to host cells. By binding to gp120, AgNPs prevent the virus from recognizing and attaching to CD4 receptors on T-cells, thereby inhibiting viral entry and replication. This mechanism highlights the potential of AgNPs in preventing HIV-1 infections by targeting early-stage viral interactions [129,130]. Methyl ester sulfonic acid (MES)-coated silver and gold nanoparticles, approximately 4 nm in size, exhibit antiviral activity against HSV-1 (*Herpes Simplex* Virus Type 1). These nanoparticles compete with the virus to bind to host cell receptors. By mimicking viral attachment sites, MES-coated nanoparticles prevent HSV-1 from interacting with host cell surface proteins, thereby blocking infection. This competitive inhibition mechanism is particularly beneficial in reducing the spread of herpesvirus infections [131]. PVP-coated AgNPs, with an average size of 69 nm ± 3 nm, interfere with viral attachment in RSV infections. RSV primarily enters host cells by attaching to surface receptors, and AgNPs can bind to viral proteins, altering their ability to attach. By disrupting this initial step of infection, AgNPs prevent RSV from gaining entry into host cells, effectively reducing viral load and transmission [132]. Both AgNPs and polysaccharide-coated AgNPs, ranging from 10–80 nm in size, have shown efficacy against the monkeypox virus. These nanoparticles block virus-host cell binding and penetration. By preventing viral adhesion to host cell membranes, they reduce the likelihood of infection. The ability of AgNPs to inhibit viral entry at multiple levels highlights their potential as broad-spectrum antiviral agents [133]. Sialic acid-functionalized gold nanoparticles (14 nm) act against influenza viruses by inhibiting viral binding to the plasma membrane. Influenza viruses rely on hemagglutinin (HA) proteins to recognize sialic acid receptors on host cells. Functionalized gold nanoparticles mimic these receptors, effectively trapping the virus and preventing it from binding to the actual host cell membrane. This mechanism significantly reduces viral infectivity and spread [134]. AgNPs and polysaccharide-coated AgNPs, around 10 nm in size, inactivate Tacaribe virus (TCRV) particles before they enter host cells. This pre-entry inactivation mechanism disrupts the structural integrity of the virus, rendering it incapable of infecting cells. Such an approach is beneficial in developing preventive antiviral strategies [121]. The development of bioactive packaging using paper coated with green-synthesized AgNPs has gained attention for its antiviral potential. Using mangosteen peel extract as a bio-reducing agent and citric acid as a crosslinker, AgNPs were synthesized at different silver nitrate concentrations. Notably, AgNPs-150 exhibited strong antiviral activity, achieving complete viral inactivation within one minute. Additionally, the coated paper demonstrated enhanced tensile strength and water resistance, making it a promising material for active packaging applications [99]. Air pollution remains a critical global issue, exacerbating health risks, especially during pandemics like COVID-19 [135]. Recent studies highlight the increasing demand for advanced filtration systems in ventilators, respirators, facemasks, and hospital air filtration. Electrospun nanofiber membranes, due to their high surface area, interconnected pores, and tunable properties, have emerged as effective materials for capturing viruses, bacteria, and pollutants. However, nanofibers alone lack intrinsic antiviral activity, necessitating surface modifications or antimicrobial agents to enhance their efficiency. Research focuses on optimizing nanofiber morphology, surface charge, and wettability, alongside developing bio-based, biodegradable, and intelligent filters that selectively trap harmful particles while reducing environmental impact [136]. Seaweed-based biological synthesis has shown promising antiviral activity against HSV-1 and HSV-2. Characterized by UV-Vis, FTIR, XRD, and TEM, the nanoparticles are spherical, ranging from 8 to 27 nm. Cytotoxic assays on Vero cells revealed an ID_50_ of 2.5 µL, demonstrating notable antiviral potential [23]. Bacillus species facilitated biological synthesis targeting the Bean Yellow Mosaic Virus. The nanoparticles, analyzed through UV-Vis, EDX, TEM, DLS, and FTIR, appeared in triangular, hexagonal, and spherical shapes, sized 77–92 nm. Seed assays on Vicia faba confirmed virus inhibition, supported by RT-PCR and ELISA analysis [137]. Bio-reduction methods synthesized nanoparticles targeting *Bombyx mori* Nuclear Polyhedrosis Virus (BmNPV). With hexagonal morphology (0.87–1.2 µm), characterized by HR-SEM, EDAX, TEM, and AFM, they interacted with the virus membrane. Silkworm assays using SDS-PAGE and energy dispersive analysis confirmed antiviral effects [25]. Chemically synthesized citrate-stabilized AgNPs, tested against Feline Calicivirus, displayed spherical structures (10, 75, and 110 nm). Characterized by SEM, mass spectroscopy, and DLS, they demonstrated viricidal and cytotoxic properties across infectivity, Western blot, and SDS-PAGE assays on FCV strain 2280 and CRFK cells [26]. Non-surface capped AgNPs synthesized chemically targeted the Vaccinia virus. TEM and XRD analysis confirmed spherical particles (25 nm ± 10 nm). In vitro assays on VERO 76, BS-C-1, and HeLa cells indicated interference in viral entry through macropinocytosis, verified by plaque, beta-galactosidase, and Western blot assays [27]. Green synthesis using *Portulaca oleracea* aqueous leaf extract produced spherical AgNPs (5–40 nm), effective against Hepatitis A virus (HAV) and Coxsackie B virus (Cox-B4). UV-Vis, FTIR, XRD, TEM, DLS, EDX, and zeta potential analysis confirmed antiviral activity, reducing viral replication and inducing oxidative stress in Vero cells with MIC values of 6.25–12.5 µg/mL [28]. UV-irradiated photochemical in situ synthesis produced AgNP-PVB nanocomposite coatings (15–118 nm). XRD, Raman spectroscopy, AFM, UV-Vis-NIR, and contact angle analysis confirmed structured spherical particles embedded in the polymer matrix. These coatings showed complete inhibition of SARS-CoV-2 at 1000 ppm (Ct > 32), validated by qRT-PCR and surface energy analysis [32]. In situ growth of AgNPs on plant fibers, using polydopamine (PDA) and glucose reduction, created well-distributed spherical nanoparticles. Analyzed by XPS, XRD, SEM, and ICP-MS, they effectively inactivated MS2 bacteriophage through plaque reduction assays. The nanoparticles interacted with viral surface proteins, enhancing antiviral and antibacterial performance with AgNP loading between 0.36–0.47 g/g [33]. The polyol method has been extensively employed to synthesize PVP-coated AgNPs, carbon-coated AgNPs, and bovine-serum AgNPs. Characterized by TEM, STEM, UV–Vis, and EDS, these nanoparticles demonstrated spherical morphologies, with sizes of 6.53 nm, 16.19 nm, and 2.08 nm, respectively. Studies by (Makhlof et al.) revealed that these AgNPs effectively interacted with the glycoproteins of HIV-1 in MT-2 and cMAGI cells, resulting in significant viral inhibition at a concentration of 25 µg/mL [106]. Tannic acid-mediated synthesis has also demonstrated antiviral efficacy, producing hexahedron-shaped AgNPs ranging from 70 to 90 nm, confirmed through TEM analysis. Butler et al. conducted cytotoxicity tests on HeLa cells, combining MTT assays, immunofluorescence analysis, and RT-PCR, which revealed that these nanoparticles interacted with adenovirus type 3 DNA, inhibiting viral replication at 9.3 µg/mL [107]. Citrate, PVP, and hydrogen peroxide-based synthesis have yielded spherical AgNPs (20–25 nm), which exhibited antiviral activity against bovine herpesvirus-1 (BoHV-1). Characterized via TEM, UV–Vis, and zeta potential analysis, these nanoparticles demonstrated anti-BoHV-1 effects in MDBK cells. The study by Abdulsattar et. al, highlighted their ability to bind viral glycoproteins, disrupting normal viral functions and reducing cytopathic effects at a concentration of 24 µg/mL [108]. Uncoated and polysaccharide-coated AgNPs synthesized for Tacaribe virus (TCRV) inhibition displayed spherical structures of 10 and 25 nm, confirmed through TEM imaging. According to Elechiguerra et al., viral inhibition assays and S-segment real-time PCR conducted on Vero cells demonstrated that these nanoparticles effectively inhibit early-stage viral replication at 25 µg/mL, with notable effects even in post-infection treatments [138]. Chitosan-stabilized AgNPs, approximately 14 nm in diameter, have exhibited potent antiviral activity against the African swine fever virus (ASFV). TEM and UV–Vis characterization confirmed nanoparticle formation, while cytotoxicity assessments on primary porcine alveolar macrophages (PAMs) by Chen et al., demonstrated significant antiviral effects at a remarkably low concentration of 0.78 ppm [139]. PVP-coated AgNPs, ranging from 8 to 12 nm, have also been effective against respiratory syncytial virus (RSV). Characterized by SEM and TEM, these nanoparticles showed activity in A549 and HEp-2 cell lines. Using qRT-PCR assays, ELISA, and plaque analysis demonstrated that the nanoparticles interacted with viral glycoproteins, preventing fusion and reducing airway obstruction at a concentration of 50 µg/mL [140].

## 5. Safety of AgNPs

AgNPs are widely used in various products for their antibacterial properties, but studies show they can be toxic to mammalian cells (Table 3). While green synthesis methods using plant extracts are preferred due to their eco-friendly nature, AgNPs can become toxic at concentrations above the LOAEL, leading to potential health risks. Future research should focus on understanding the factors influencing AgNP toxicity and the mechanisms behind it to minimize environmental and human health impacts while utilizing their beneficial properties [141]. Understanding toxicity mechanisms is crucial for their safe use in the future [142]. Recent research on AgNPs highlighted their antibacterial, antiviral, and anticancer properties, but concerns about their potential toxicity remain. While AgNPs are used in various products, including medical and consumer goods, their safety is a major focus. Studies emphasize the need for safer synthesis methods, such as green synthesis, and the evaluation of their biocompatibility and cytotoxicity. In-depth research is essential to ensure their safe use in therapeutic applications [143]. For instance, a study by Alwan et al. evaluates the safety of biosynthesized AgNPs using *Cinnamomum zeylanicum* bark extract in rats. After 14 days of oral administration at varying doses, no significant toxicity was observed. There were no changes in body weight, biochemical markers (AST, ALT, urea, creatinine), oxidative stress parameters (Mass drug administration (MDA), Superoxide Dismutase (SOD), catalase (CAT)), or histopathological features of the liver and kidneys, suggesting that AgNPs are relatively safe at the tested doses [144]. Another study by Vuković et al. investigated the safety of AgNPs with different surface coatings on the human immune system. Four types of AgNPs were tested for their effects on human peripheral blood mononuclear cells (hPBMC). Results showed that AgNPs, particularly positively charged and protein-coated ones, induced apoptosis, cell death, oxidative stress, and mitochondrial damage. The study highlighted the genotoxic potential of AgNPs, providing valuable insights for assessing the safety of nanosilver in medical applications [145]. The toxicity of AgNPs also depends on the organism’s defense mechanisms and the culture media used in testing. AgNPs and released Ag^+^ cause toxicity by damaging membranes, generating reactive oxygen species (ROS), and inducing protein oxidation, mitochondrial dysfunction, DNA damage, and cell proliferation inhibition. AgNPs interact with sulfur-containing macromolecules, contributing to toxicity. Their antibacterial activity and ability to penetrate cell membranes lead to cytoplasmic accumulation, oxidative damage, and apoptosis in mammalian cells [146]. Elyousfi et al. evaluated the ecotoxicity of silver nanoparticles (Ag NPs) on *Ruditapes decussatus* by analyzing biochemical changes in gills and digestive glands after exposure to 100 and 200 μg concentrations for 48 h and 7 days. AgNPs disrupted antioxidant and cholinergic systems, with time- and concentration-dependent effects on catalase (CAT) and glutathione S-transferase (GST) activities. Acetylcholinesterase (AChE) activity decreased, especially at higher concentrations, suggesting significant toxicity [147]. Pinheiro et al. investigated the toxicity of AgNPs in *Artemia salina* linked to the interaction between silver ions (Ag^+^) and chitin in the organism’s cuticle. AgNPs at concentrations of 50 and 100 ppm caused mortality and cellular damage after 24–48 h. Geometric optimization and SAPT0 analysis revealed that Ag3+ ions deform the chitin structure. Light and confocal microscopy confirmed AgNPs’ presence in the cuticle and the resulting cellular damage, shedding light on the toxicity mechanism [148]. Baloushi et al. synthesized AgNPs using *Moringa peregrina* leaf extract and demonstrated significant antioxidant and anticancer activity against human cancer cell lines (Caco-2 and MCF-7). These nanoparticles are eco-friendly and non-toxic to humans, showing promise for biological applications such as drug delivery and disease treatment. However, the study did not assess the effects on normal cells, highlighting the need for further research on AgNP toxicity, surface modifications, and underlying bioactive mechanisms [149]. Silver nanoparticles (Ag NPs) at a dose of 21.5 mg/kg effectively treated *Trichinella spiralis* infection in mice, showing high efficacy against adult and encapsulated larvae. The Ag NP treatment, either alone or combined with multivitamins (MM), achieved significant trichinocidal effects, surpassing reference drugs. Importantly, combining Ag NPs with MM alleviated silver-induced toxicity, improving redox parameters and liver and kidney biomarkers, thus overcoming the adverse effects of silver material while maintaining therapeutic effectiveness [150]. AgNPs exhibit concentration-dependent toxicity across biological systems. In a study, Dinç assessed their effects on *Escherichia coli*, *Bacillus subtilis*, *Caenorhabditis elegans*, and human vein endothelial cells (HUVECs). AgNPs inhibited bacterial growth (52% at 50 µg/mL), reduced *Caenorhabditis elegans* reproduction by 25% at 10 µg/mL, and caused a significant decrease in body bending frequency. HUVECs showed cytotoxicity, with an IC_50_ of 38 µg/mL. Findings emphasized the importance of cautious AgNP application in biomedical and environmental fields [151]. Kakakhel et al. found that Long-term exposure to high concentrations of AgNPs led to toxicity, bioaccumulation, and tissue damage in common carp (*Cyprinus carpio*). Fish exposed to AgNPs showed accumulation primarily in the liver, followed by the intestine, gills, and muscles. Histological alterations, including necrosis and tissue degeneration, were observed, particularly at higher concentrations [152]. Thwala et al. evaluated the toxicity of AgNPs on the aquatic plant *Salvinia minima*, focusing on size, bioaccumulation, and environmental interactions. Smaller AgNPs (10 nm) exhibited greater solubility, accumulation, and toxicity than larger ones (40 nm). Exposure reduced plant growth, chlorophyll content, and overall health, with toxicity influenced by water chemistry [153]. Polystyrene nanoplastics influence the toxicity of AgNPs in zebrafish embryos by acting as carriers in water. The release of silver ions (Ag^+^) from AgNPs was 4.23%. While AgNPs altered antioxidant and metabolic gene expression, nanoplastics mitigated apoptosis and immunotoxicity. Findings by Yan et al. suggested nanoplastics reduce AgNP genotoxicity by absorbing Ag^+^ and forming aggregates, highlighting the complex interactions between pollutants in aquatic environments [154]. AgNPs supported on zirconium dioxide (ZrO_2_) modified with dihydroquercetin (DHQ) exhibited strong antibacterial effects against *Escherichia coli* and *Staphylococcus aureus*, with complete bacterial reduction at all tested Ag concentrations. However, cytotoxicity tests on HeLa and MRC-5 cells showed minimal toxicity, even at high concentrations. This suggests that the modified AgNPs provide effective antimicrobial properties while maintaining biocompatibility, highlighting their potential for biomedical applications [155]. A study by Sambale et al. examined the toxic effects of AgNPs on mammalian cell lines, including human liver, lung, and fibroblast cells. Results show that AgNPs significantly reduce cell viability, with smaller particles exhibiting higher toxicity. The toxicity is attributed to nanoparticle interaction rather than silver ions alone. AgNP exposure triggers apoptosis rather than necrosis [156]. AgNPs synthesized using entomopathogenic fungi show antimicrobial properties and potential use as nanoinsecticides. However, toxicity studies are essential to assess their environmental impact and safety. AgNPs may cause cytotoxicity and genotoxicity, depending on size, shape, and concentration. In vitro methods like MTT and comet assays help evaluate their effects on cells [157]. Greulich et al. studied the toxicity of AgNPs and silver ions in bacteria and human cells. It was found that the toxic effects occur within similar concentration ranges, challenging the assumption that silver is significantly safer for mammalian cells. Both forms of silver affect *Escherichia coli*, *Staphylococcus aureus*, mesenchymal stem cells, and blood cells through ion release and reactive oxygen species. The findings raise concerns about the widespread use of silver in medical and consumer applications due to potential human toxicity [158]. Cho et al. examined the acute toxicity of AgNPs in mice, focusing on size-dependent effects. Mice administered 10 nm AgNPs exhibited reduced activity, body temperature drop, and organ damage, including liver necrosis and spleen congestion. Larger AgNPs (60 and 100 nm) showed significantly lower toxicity. Findings suggest smaller nanoparticles pose greater health risks due to higher reactivity and absorption, raising concerns about their widespread use in consumer and medical products [159]. Jian et al. investigated the toxicity of AgNPs against the fungus *Fusarium graminearum*, which produces harmful mycotoxins. AgNPs effectively inhibit fungal growth by damaging cell membranes, impairing metabolism, and increasing oxidative stress. However, they also trigger the production of deoxynivalenol (DON), a dangerous mycotoxin. Despite their antifungal potential, AgNPs pose risks of enhanced toxin production, necessitating careful evaluation before agricultural application to prevent unintended health and environmental hazards [160]. Similarly, Zhao et al. investigate the toxic effects of AgNPs on *Chlamydomonas reinhardtii*, a freshwater microalga. Exposure to AgNPs inhibits growth, damages chloroplasts, reduces photosynthetic pigment production, and increases oxidative stress. The nanoparticles disrupt membrane integrity, leading to increased permeability and cellular damage. Activation of antioxidant enzymes was observed as a defense mechanism. These findings highlight potential environmental risks of AgNP contamination in aquatic ecosystems due to their toxicity to primary producers [161]. The study by Souza et al. evaluated the toxicity of AgNPs on the aquatic plant Lemna minor, focusing on solubility, accumulation, and size-dependent effects. Smaller AgNPs (30 nm) showed higher solubility, accumulation in roots and leaves, and greater toxicity, causing 60% mortality at high concentrations. Larger AgNPs (85 and 110 nm) had lower toxicity [162]. Ke et al. investigate the toxic effects of AgNPs on *Arabidopsis thaliana*, focusing on their impact on flowering and offspring development. AgNP exposure reduced petal and pollen viability, delayed flowering, and impaired seed production. The toxic effects were transferred to offspring, leading to worsened plant growth and delayed flowering. Gene expression related to floral organ development was downregulated, highlighting potential risks to plant reproduction and food security due to nanoparticle contamination [163]. Marinho et al. examined the toxicity of AgNPs in *Danio rerio* (zebrafish) by analyzing their effects on brain, muscle, liver, and gill tissues. Exposure to AgNPs reduced acetylcholinesterase activity in the brain and muscle, inhibited catalase in the liver and gills, and caused morphological damage in gills, including lamellar fusion and epithelial lifting [164]. Maziero et al. evaluated the toxicity of AgNPs stabilized with gum arabic protein (AgNP-GP) in different species, including *Daphnia similis*, *Danio rerio* embryos, and Sprague Dawley rats. AgNP-GP caused significant toxicity in aquatic organisms, leading to immobility in *Daphnia similis* and developmental defects in zebrafish embryos. However, oral administration in rats up to 10 mg/kg for 28 days showed no adverse effects. These findings highlight species-specific toxicity concerns of AgNPs [165]. Abdelkhaliq et al. examined the potential developmental toxicity of AgNPs using the BeWo b30 placental transport model and the embryonic stem cell test (EST). Findings show that AgNPs can cross the placental barrier, but their transport is limited and influenced by surface chemistry. While AgNPs exhibit cytotoxicity, they do not induce developmental toxicity at non-cytotoxic concentrations. Aged silver sulfide nanoparticles (Ag2S NPs) demonstrate lower toxicity and bioavailability [166]. Chen et al. investigated the immunotoxicity of AgNPs using a zebrafish model. Exposure to AgNPs caused mortality, malformations, and immune system toxicity, affecting neutrophils and macrophages. AgNPs also disrupted immune-related gene expression and increased oxidative stress. However, pterostilbene (PTE), a natural antioxidant, reduced these toxic effects by activating immune cells and mitigating oxidative stress. The findings highlighted the potential immune risks of AgNPs and the protective role of PTE [167]. Ajdary et al. investigated the toxicity of AgNPs on endometrial receptivity in female mice. Mice exposed to AgNPs (2 and 4 mg/kg) showed increased inflammatory markers (IL-6, IL-1β), nanoparticle accumulation in endometrial tissue, and reduced pinopod and microvillus formation, affecting implantation. This suggested that AgNP exposure during pregnancy may disrupt uterine conditions, highlighting potential reproductive risks and the need for caution in nanoparticle exposure during gestation [168]. Emma et al. evaluated the sub-acute and chronic toxicity of AgNPs synthesized using *Azadirachta indica* extract in Swiss albino rats. While no mortality or significant weight changes were observed, higher doses (30 mg/kg) led to increased liver enzymes (ALT, AST) and portal hepatitis. Kidney function remained unaffected [169]. Khoshnamvand et al. examined the toxicity of biosynthesized silver nanoparticles (AR-AgNPs) on different aquatic organisms, including phytoplankton (*Chlorella vulgaris*), zooplankton (*Daphnia magna*), and fish (*Danio rerio*). Ag^+^ ions were more toxic than AR-AgNPs at all trophic levels, with Daphnia magna being the most sensitive. Toxicity mainly originated from nanoparticles rather than ion release. These findings highlight the potential ecological risks of AgNP contamination in aquatic food chains [170]. The study by Somda et al. investigated the biosynthesis, characterization, antimicrobial activity, and safety of AgNPs derived from *Brassica carinata* microgreens. AgNPs exhibited strong antimicrobial properties against various pathogens but showed minimal cytotoxicity on Vero cells, indicating potential biocompatibility [171]. Silver nanoparticles offer significant benefits but raise toxicity concerns, including oxidative stress, DNA damage, and inflammation. Their impact varies with size, concentration, and exposure duration. Comprehensive research is necessary to understand long-term effects, ensuring their safe application while minimizing potential risks to human health and environmental sustainability [172].

### Limitations of AgNPs

AgNPs offer distinct advantages due to their unique physicochemical properties when compared to bulk silver. However, they also face certain limitations that can affect their performance and effectiveness. One significant issue is their susceptibility to oxidation. AgNPs react readily with oxygen, leading to the formation of silver ions that bind with oxygen to create strong ionic bonds. This oxidation process alters the structure of the nanoparticles, thereby modifying their physicochemical characteristics. The oxidized form of AgNPs can also reduce their antibacterial efficacy, as the activity of the nanoparticles is closely linked to the presence of silver ions. Another challenge with AgNPs is their tendency to aggregate. Research shown that AgNPs are prone to aggregation when placed in organic solvents, such as dimethylformamide and tetrahydrofuran, which can impact their stability and performance in various applications.

## 6. Challenges and Future Directions

The use of AgNPs as antiviral agents holds significant promise, but several challenges remain that must be addressed to maximize their potential. Several approaches for the enhancement of the antimicrobial activity of AgNPs could be by utilizing AgNPs as antiviral agents, some aspects should be commented such as magnetic-responsive Ag NPs, light-triggered antimicrobial activity, and targeting of viral agents. Prucek et al. synthesized and characterized two magnetic silver-based nanocomposites—Ag@Fe_3_O_4_ and γ-Fe_2_O_3_@Ag—using maltose-mediated silver reduction and polyacrylate as a spacer [176]. The nanocomposites showed strong antibacterial and antifungal activity (MIC: 1.9–125 mg/L) and limited cytotoxicity in fibroblasts (toxic < 430 mg/L (Ag@Fe_3_O_4_)). Their magnetic and biocompatible properties suggest potential for targeted delivery of silver nanoparticles. In another study, Torres-Mendieta et al. achieved biofilm deterioration in bacteria using magnetic doping of AgNPs [177]. Zhang et al. reported the immobilized AgNPs (Fe_3_O_4_–SiO_2_–Ag) onto magnetic silica composite depicting an enhanced antibacterial activity [178]. A study conducted by Ratti and coworkers demonstrated that the antibacterial activity of laser-ablated AgNPs could be enhanced by irradiation with visible light [179].

One of the key challenges is understanding the precise parameters that enhance their antiviral efficacy, such as particle size, concentration, and functionalization. While smaller AgNPs (around 10 nm) have demonstrated stronger antiviral effects, increasing the particle size can reduce efficacy. Moreover, the concentration of AgNPs in the system is critical, as higher concentrations lead to a stronger antiviral effect, though excessive amounts may cause toxicity. *Functionalization* of AgNPs and their incorporation into composite materials also present opportunities for improving antiviral activity by either enhancing the particles’ interaction with viruses or by incorporating antiviral agents into the material itself. Despite these promising findings, the commercial application of AgNPs, particularly for antiviral purposes, is still limited. While products such as face masks and textiles have been introduced, more advanced systems, particularly those for treating viral infections, are mostly in the prototype stage. The most promising applications of AgNPs include their use in water and air purification systems, as well as in the textile industry, with the potential to significantly impact public health and quality of life, especially during viral pandemics like COVID-19. However, the mechanisms of AgNPs’ antiviral action remain poorly understood, and further research is necessary to optimize their performance, particularly in diverse devices and applications. Additionally, environmental concerns about the toxicity and accumulation of silver nanoparticles must be addressed. Sustainable methods for collecting or recycling AgNPs could reduce waste and production costs, making these technologies more viable. Ultimately, while the antiviral applications of AgNPs are promising, their future success hinges on overcoming these challenges through further scientific investigation into their effectiveness, safety, and environmental impact.

## Figures and Tables

**Table 1:** Green synthesis of silvern nanoparticles (AgNPs).

Plant Used	Characterization Methods	Size (nm)	Properties	Method	Application and Significance	Reference
*Lallemantia royleana*	TEM	34.47 ± 1.6	Spherical shape, antioxidant, antimicrobial, anti-inflammatory, cytotoxic	Green synthesis using leaf extract	Biocatalytic degradation of methylene blue and biopharmaceutical applications, with potential environmental and medical benefits from green synthesis using leaf extract.	[51]
*Kalanchoe fedtschenkoi*	UV-Vis, FTIR, SEM, Zeta Potential	39.9, 111, 42	Antibacterial (*Escherichia coli*, *Staphylococcus aureus*, *Pseudomonas aeruginosa*), antioxidant	Green synthesis using plant extracts	Strong antibacterial and antioxidant properties, with biomedical and nanotechnology applications, achieved through green synthesis using plant extracts.	[52]
*Rubus discolor*	UV-Vis (λmax 456.01 nm), TEM, XRD, EDX, Zeta Potential (−44.2 mV)	37	Crystalline structure, high stability, antibacterial (MDR *Escherichia coli*, *Pseudomonas aeruginosa*), cytotoxic (A431, MCF7, HepG2)	Green synthesis using leaf extract, Response Surface Methodology	Effective in medical applications and antimicrobial coatings, with phenolics, tannins, and flavonoids contributing to bioactivity, synthesized through green methods and Response Surface Methodology.	[53]
Green tea	XRD, FESEM, DLS	50	Quasi-spherical shape, antibacterial (*Staphylococcus aureus*, *E. coli*)	Green synthesis from recycled silver (radiographic films)	Sustainable nanoparticle production and antimicrobial coatings, utilizing recycled silver from radiographic films for eco-friendly synthesis.	[54]
*Malachra alceifolia*	UV-Vis, XRD, SEM	10–55 (avg. 28)	FCC crystalline structure, antibacterial *Pseudomonas aeruginosa*, *Staphylococcus aureus*, antioxidant (DPPH scavenging, IC_50_: 0.87 mg/mL)	Green synthesis using leaf extract	Biomedical, antimicrobial, and antioxidant applications, with strong activity attributed to green synthesis using leaf extract.	[55]
*Curcuma amada*	Not specified	Not specified	Various potential applications	Surfactant-free, eco-friendly synthesis using rhizome essential oil	Eco-friendly synthesis using rhizome essential oil, focusing on biomedical and nanotechnology applications with a sustainable approach.	[56]
*Paullinia cupana Kunth* (Guarana)	UV-Vis, DLS, Zeta Potential, MET, NTA, EDX	39.33–126.2	Spherical morphology, high colloidal stability, antibacterial, antioxidant, cytotoxic against cancer cells, effective against *Leishmania*	Green synthesis using aqueous leaf extract	Sustainable AgNP synthesis with diverse applications in biomedical, environmental, and industrial fields. Seasonal extract variation enhances properties.	[57]

**Table 2:** An updated summary of some important antiviral studies on AgNPs.

Sr.No	Method of Synthesis of AgNPs	Type of Virus/Pathogen	Characterization of AgNPs	Shape of AgNPs	Size of AgNPs	Viral/Bacterial/Disease Model	Assays/Evaluation Parameters	Mechanism of Action (MOA)	Doses	References
1	Sputtering deposition on polylactide (PLA) films	Herpes simplex virus type 1, Influenza A virus	WAXS, TEM, TGA, DSC	Not specified	5.9 nm	In vitro: MDCK, BHK, Hep-2 cells	Antimicrobial, antiviral, and cytotoxicity assays	Weak virucidal effect, not cytotoxic	Not specified	[88]
2	Oleylamine-capped AgNPs deposited on nonwoven textile	SARS-CoV-2	FTIR, DLS, TEM, ICP-OES	Spherical	8 ± 2 nm	In vitro: Virus inactivation on textile surface	Virus inactivation assay (99.6% in 2 min)	Direct virucidal activity	Microgram amounts	[89]
3	Direct pulmonary administration of AgNPs	Influenza virus, Murine pneumonia virus	Not specified	Not specified	Not specified	In vivo: Mice model	Viral load reduction, cytokine analysis, immune response evaluation	Enhances NK cell migration and IFN-γ production	Not specified	[90]
4	Green tea extract-mediated synthesis	Foot-and-mouth disease virus (FMDV)	TEM, FTIR, DLS	Spherical	15.1–16.9 nm (TEM), 28.86 nm (DLS)	In vitro: BHK-21 cells	TCID_50_, cytopathic effect inhibition assay, IC_50_, SI	Suppresses viral replication in early stages	IC_50_: 2.05–2.45 µg/mL	[91]
5	In situ functionalization of clinoptilolite with AgNPs using tannic acid	Bacterial pathogens (Gram-positive and Gram-negative)	FTIR, TEM, Compositional analysis	Spherical	2–4 nm	Not virus-specific (Bacterial model)	Zone of inhibition test	Antibacterial activity via surface adsorption	Not applicable	[92]
6	Green synthesis using *Trichoderma reesei* fungus	SARS-CoV-2	UV-Vis, TEM, SEM, DLS, Zeta Potential	Spherical	7–50 nm (TEM), 86.74 nm (DLS major)	In vitro: Vero E6, Calu-3 cells; In vivo: Syrian hamsters	Cytotoxicity (MTT), RT-qPCR, Immunohistochemistry, Inflammasome Activation	Binds spike protein, reduces replication, modulates immune response	Not specified	[93]
7	Green and chemical synthesis using *Citrus limon* extract	Bovine mastitis pathogens (*Staphylococcus aureus*, *E. coli*)	UV-Vis, Electron Microscopy, Zeta Sizing, FTIR, GC-MS	Not specified	10–20 nm	In vitro: Antimicrobial testing	MIC_50_ (46.10 and 49.93 µg/mL for green AgNPs), MIC_50_ (77.39 and 86.50 µg/mL for chemical AgNPs)	Disrupts bacterial cell viability	Not specified	[94]
8	Green synthesis using *Telfairia occidentalis* leaves and stems	Not virus-specific (Anti-inflammatory, Anti-diabetic, Antioxidant applications)	UV-Vis, FTIR, SEM	Spherical	10–80 nm (SEM), 43.66 nm mean	In vitro: Antioxidant, anti-diabetic, and anti-inflammatory assays	α-glucosidase inhibition, protein denaturation inhibition, radical scavenging assays	Phytochemicals stabilize and reduce AgNPs, inhibit enzymes, and neutralize free radicals	Not specified	[95]
9	Green synthesis using *Cuscuta epithymum* extract	Not virus-specific (Antioxidant, Antibacterial, Antitumor properties)	UV-Vis, FESEM, TEM, XRD, FTIR	Spherical	15–60 nm	In vitro: Antioxidant, antibacterial, and cytotoxicity assays (MCF-7 cells)	DPPH assay (IC_50_ = 45.55 mg/L), Disk Diffusion for antibacterial activity, MTT cytotoxicity assay (IC_50_ = 42.53 mg/L, 36.78 mg/L, 26.86 mg/L at 12, 24, 48 h)	Reduction of Ag^+^ ions to AgNPs, oxidative stress modulation, antibacterial and antitumor activity	Not specified	[96]
10	Deep eutectic solvent (DES) method using betaine, glucose, and ethylene glycol	Influenza A/H1N1, Human Coronavirus (HCoV-OC43), *Vesicular Stomatitis* Virus (VSV)	STEM, XPS, DLS, UV-VIS	Not specified	50–100	Human Influenza A/H1N1, HCoV-OC43 (Betacoronavirus 1), VSV (Rhabdoviridae)	ROS generation assays, API assays, MIC/MBC determination, Cell decomposition rate assays	ROS generation leading to enzyme inactivation and inhibition of metabolic processes	Virus titer reduction of 93.7−99.96%	[97]
11	Fungal-mediated synthesis using *Cephalosporium aphidicola* (eco-friendly approach)	Not specified	UV-Vis, FT-IR, EDX, FE-SEM, DLS	Spherical	59.52	Not specified	Antibacterial and biofilm degradation assays, DPPH radical scavenging, Alpha-amylase inhibition, Urease inhibition	Antimicrobial, biofilm degradation, enzyme inhibition, antioxidant activity	1 mg/mL (72.81% DPPH scavenging, 86.06% alpha-amylase inhibition, 80.84% urease inhibition)	[98]
12	Green synthesis using *Trema orientalis* (L.) leaf extract	Not specified	UV-Vis, FTIR, XRD, TEM, AFM	Spherical, Crystalline	14.04–34.38 (avg. 26.81)	Not specified	Antibacterial assay (Agar well diffusion), MIC	Flavonoids mediate Ag^+^ reduction and stabilization, leading to antibacterial activity	MIC_50_ = 55.31 μg/mL; Zone of inhibition: 9, 10, 13, 14 mm at 25, 50, 75, and 100 µg/mL	[48]
13	Green synthesis using *Garcinia mangostana* (GM) peel extract and citric acid for active packaging	Not specified	UV-Vis, Elemental analysis, Silver mapping	Spherical	2.36–294.73	Not specified	Virus inactivation assay, Antibacterial (*E. coli*, *Staphylococcus aureus*), Water resistance, Tensile strength analysis	Surface roughness increased hydrophobicity, synergistic effect of AgNPs, citric acid, and GM extract	AgNPs-150 coated paper showed complete virus inactivation within 1 min	[99]
14	Silver nanoparticles (AgNPs) (20 mg/mL) coated with natural resins from Noble Elements LLC	Influenza A (H1N1), strain A/FM/1/47	Size: 10 ± 1.5 nm, Stable dispersion, Natural resin coating	Likely spherical (inferred from uniform dispersion)	10 ± 1.5	MDCK cells, H1N1 virus (1 × 10^7^ TCID_50_/mL)	MTT and Neutral Red (CC_50_ = 80 μg/mL), Virucidal activity, Pre- and Post-exposure assays, Infective titer assay	Prevents viral attachment, disrupts envelope, inhibits replication. Selective Index: Pre-exposure = 88, Post-exposure = 667 (higher than oseltamivir)	0.0002 to 100 μg/mL tested. Effective concentrations: Pre-exposure = 4.5 μg/mL, Post-exposure = 0.6 μg/mL	[100]
15	Green synthesis using *Nigella arvensis* aqueous extract	HSV-1, HAV, Adenovirus	UV-Vis, XRD, TEM	Spherical	2–9 (avg. 2.5)	In vitro cell culture	Antiviral efficacy assay, MIC/MBC determination, Color change (yellow to brown)	Inhibits viral replication by 53.6% (HSV-1), 86% (HAV), 17.3% (Adenovirus)	MNTC: 10.56 µg/mL; MIC: 5.7–10.2 μg/mL; MBC: 22.3–36.8 μg/mL	[101]
16	Chitosan nanoparticles (CS-NPs) and chitosan silver nanocomposites (CS-Ag NC)	*Alfalfa mosaic virus* (AMV) in pepper plants	Electron microscopy	Spherical	Uniform	AMV-infected pepper plants	ELISA, Symptomatology, RT-PCR, Agronomic metrics (plant height, fresh and dry pod weight, number of pods)	Induces phenol, proline, and capsaicin production; inhibits AMV replication	400 ppm CS-NPs (90% inhibition), 200 ppm CS-Ag NC (91% inhibition)	[102]
17	Polyvinylpyrrolidone (PVP)-stabilized AgNPs	Spring viraemia of carp virus (SVCV), European catfish virus (ECV), Ictalurid herpesvirus 2 (IcHV-2)	TEM, DLS	Spherical, Electron-dense	10.2 ± 1.6 (TEM), 22.4 ± 5.3 (DLS)	Fish viruses in EPC cells	Virus pretreatment, Cell pretreatment, Cell post-treatment, Delayed post-treatment (24 h after infection)	Inhibits viral replication, disrupts viral envelope, prevents host cell binding	25 ng/mL (safe concentration), Reduction in viral load: 70–330× (ECV), 10–54× (SVCV), 5–17× (IcHV-2)	[103]
18	Green synthesis using *Punica granatum* biowaste peel extract	Tobacco mosaic virus (TMV)	SEM, TEM, UV-Vis, XRD, DLS, EDX, FTIR, Zeta potential	Spherical, Condensed	61–97 (SEM), 33.37 ± 12.7 (TEM)	TMV-infected tomato plants	Greenhouse study (TB, TA, TD treatments), PR gene expression, Oxidative stress markers, Antioxidant enzyme assays	Reduces viral accumulation, delays viral replication, enhances PR gene expression, restores flavonoid biosynthesis	TD strategy (dual treatment) most effective	[104]
19	Biological synthesis using fungi	Herpes Simplex Virus and Human Parainfluenza Virus Type 3	TEM, UV-Vis, zeta potential	Spherical	46 nm and 40 nm	VERO cells	MTT assay, cotreatment assay, cell pretreatment assay, cell post-treatment assay, Virus pretreatment assay	Inhibits viral replication	ID_50_-10 mg/mL,	[105]
21	Green synthesis using *Ulva lactuca* extract; AgNO_3_ (4 mM) + algal extract (5:5) at 60 °C under light for 84 h	Adenovirus Type 2	UV-Vis, TEM, XRD, SEM	Spherical and distinct AgNPs	4.08–27.57 nm (avg. 10.29 nm)	In vitro: Vero cell line (African green monkey kidney cells)	MTT (cytotoxicity), Plaque Reduction, TCID_50_ (viral infectivity)	Weak antiviral activity via possible inhibition of viral entry or replication. Lower activity than Amantadine	2–3000 µg/mL tested. CC_50_ = 20.34 µg/mL, 9.83% inhibition at 2 µg/mL	[106]
22	Hydroxylamine-reduced Ag colloidal nanoparticles (AgNO_3_ + NH_2_OHHCl, 350 rpm, 45 min)	Not applicable (focus on antiviral drug detection)	UV-Vis, DLS (size and distribution)	Spherical	56.42 nm (average)	Not applicable (focus on Tenofovir (TFV) detection)	SERS, PLS Regression, CHAOS Theory-based Spectral Ranking	AgNPs serve as a SERS substrate, enhancing Raman signals for ultra-sensitive TFV detection, aiding in HIV drug adherence monitoring	TFV detection down to 25 ng/mL, using double-deposition for enhanced sensitivity	[107]
23	Green synthesis using *Taraxacum officinale* (dandelion) root extract; AgNO_3_ (0.315 g in 100 mL) reduced under alkaline conditions (pH 10) with NaOH, followed by microwave heating	SARS-CoV-2	XRD, FTIR, FESEM	Spherical	15–60 nm (FESEM), 11–22 nm (XRD crystallite size)	In vitro: WI-38 human lung fibroblast cells infected with SARS-CoV-2	XTT assay (cell viability), Plaque Reduction, Microscopic analysis for viral inhibition	Blocks viral entry, disrupts viral proteins, inhibits replication; Alcoholic extract showed stronger antiviral activity due to smaller particle size	50, 25, and 10 mg/L tested. IC_50_: 32.50 mg/L (alcoholic), 29.03 mg/L (aqueous)	[108]
24	Green synthesis using *Alocasia odora* rhizome (RE) and stem extract (SE); AgNPs synthesized from aqueous extracts	Dengue virus type 2 (DENV-2)	UV-Vis, SEM, EDX, FTIR	Spherical	RNP: 60.83–64.66 nm, SNP: 54.64–149.06 nm	In vitro: Huh-7 cell line infected with DENV-2	MTT assay (cytotoxicity), Plaque Reduction, Microscopic analysis for cytopathic effects (CPE)	SNP and RNP significantly reduce viral infectivity titer; SNP shows stronger cytopathic effects against DENV-2	12.5 µg/mL: SNP reduces virus-infected cells by 73% ± 2.64, RNP by 70% ± 5	[109]
25	Green synthesis using *Solanum mammosum* (*Sm*) leaf extract; AgNPs and AuNPs synthesized using NaBH_4_ as a reducing agent	PhiX174 (non-enveloped) and Phi6 (enveloped) bacteriophages (surrogate models for SARS-CoV-2)	UV-Vis, TEM, FTIR, HPLC-DAD	Spherical	AuNPs-Sm: 5.34 ± 2.25 nm, AgNPs-Sm: 15.92 ± 8.03 nm	In vitro: Phi6 with *Pseudomonas syringae* host, PhiX174 with *Escherichia coli* host	Antiviral assay (viral inactivation), Cytotoxicity assays (A549, HFF cell lines)	AgNPs-Sm and AuNPs-Sm inactivate Phi6, likely by interacting with viral envelope proteins; AgNPs are more effective than AuNPs	AgNPs-Sm: 99.94% viral inactivation at 0.01 mg/mL, AuNPs-Sm: 99.30% at 1 mg/mL	[110]

**Table 3:** Toxicity effects of Silver Nanoparticles.

S. No	Method of Preparation	Shape and Size	In Vitro/In Vivo	Toxicity and Effects	References
1	Co-precipitation using silver nitrate and trisodium citrate	Spherical, ~25 nm, <40 nm thick	In vitro: Tested on HepG2 and lung cells (IC_50_ measured) In vivo: Infected mice treated orally; organ biomarkers (ALT, AST, urea, creatinine) assessed	Mild liver and kidney toxicity; reversible with multivitamins. Strong antiparasitic effect and reduced oxidative stress when combined with supplements.	[150]
2	Commercial AgNP colloid (Nanocid^®^) TiO_2_: Powder suspended and sonicated	Spherical, AgNPs: Avg 7.29 nm TiO_2_: ~32.3 nm	In vivo only: Common carp exposed to AgNPs, TiO_2_NPs, or their mixture. Histology, bioaccumulation, enzyme activity, and growth performance assessed	Co-exposure increased toxicity. Caused gill tissue damage, reduced antioxidant enzymes, increased silver bioaccumulation in liver/intestine, and reduced weight gain.	[173]
3	Green synthesis using microgreen extract (BCME) + AgNO_3_ under heat and stirring	Spherical, avg. 34.68 nm Crystalline structure confirmed	In vitro only: Cytotoxicity tested on Vero cells using MTT assay	Low cytotoxicity on Vero cells. Safe for biological use and exhibited strong antimicrobial activity.	[171]
4	Green synthesis using “Katti Peptide” + gum arabic protein	Core: 20 ± 5 nm (TEM); Hydrodynamic: 70–80 nm	In vitro: Daphnia similis, zebrafish embryos In vivo: Sprague Dawley rats	EC_50_ (Daphnia): 4.4 μg/L LC_50_ (Zebrafish): 177 μg/L No adverse effects in rats up to 10 mg/kg	[165]
5	Green synthesis (method not detailed)	Not specified	Plant-based (In vivo)	AgNPs improved growth under Pb stress Reduced oxidative damage; enhanced chlorophyll, antioxidants	[174]
6	Aqueous leaf extract of *Moringa peregrina*	Spherical; 18–27 nm (HR-TEM)	In vitro: MCF-7 and Caco-2 cell lines	IC_50_: 26.93 μg/mL (MCF-7) IC_50_: 41.59 μg/mL (Caco-2) Good antioxidant and anticancer activity	[149]
7	Green synthesis using methanolic bark extract of *Azadirachta indica*	Spherical; ~45 nm (SEM)	In vivo: Swiss albino rats	Sub-acute (28 days) and chronic (180 days): No major toxicity up to 10 mg/kg Liver damage at 30 mg/kg	[169]
8	Methods not detailed in the document	Spherical; ~2 nm	In vitro (fungal cultures)	Effective against resistant strains Induced ROS, DON production Cellular damage observed	[160]
9	Pre-synthesized spherical AgNPs	Spherical, 18–30 nm	In vivo: Female NMRI mice	4 mg/kg dose led to increased IL-6, IL-1β, reduced pinopods Nanoparticle accumulation in endometrium	[175]
10	Commercial AgNPs with coatings (lipoic acid, citrate, BPEI), Ag_2_S	Spherical; 45–51 nm (TEM)	In vitro: BeWo b30 cell layer + Mouse embryonic stem cell test	Pristine AgNPs slightly cross placenta; embryotoxicity observed only at cytotoxic levels; Ag_2_S least toxic	[166]
11	Chemical reduction (NaBH_4_ and AgNO_3_)	Spherical; 55 ± 7 nm	In vitro: HUVECs, bacteria In vivo: C. elegans	Toxic to all systems in a dose-dependent manner; IC50 (HUVECs): 38 μg/mL; reduced motility and reproduction in *C. elegans*	[151]
12	Green synthesis using entomopathogenic fungi	Not specified	In vitro (MTT, Comet assays, pest control bioassays)	Toxic to pests; good antimicrobial activity; low cytotoxicity; potential for eco-friendly biopesticide	[157]

## Data Availability

Data used in the preparation of this review article is already available as published papers in the literature.
